# Mauser Bullet Wounds

**Published:** 1900-02-17

**Authors:** 


### MAUSER BULLET WOUNDS.

From the description given by various medical observers at the seat of war it is evident that much which has hitherto been accepted in regard to the characteristics of bullet wounds will require considerable modification. In old days much used to be made of the manner in which bullets became diverted from their course by slight obstacles in their path, and tales are to be found in plenty describing how bullets which, from the position of the entrance and exit wounds, appeared to have gone right through the body had really run round under the skin, doing but little damage. The modern bullet is very different. From its small size and its high velocity, except when spent—a condition rarely met with in the fighting line—it is hardly ever materially diverted from its course in its passage through the body, even through it may traverse the body from head to foot. Thus the same bullet may and often does cause quite a number of separate injuries, traversing in some cases a series of viscera, and in others different limbs, or limb and trunk one after the other, as often occurs when the limbs are flexed as in kneeling, or when the arm is bent as in firing. Again, the frequency with which wounds are received when lying down introduces a peculiar injury in the form of bullet tracks running through the body lengthwise. What is very interesting in regard to many of the wounds met with is the small calibre, or section, of the injured area around the bullet track, only the tissue immediately surrounding the track of the bullet being condensed and contused, so that structures lying but a very short distance on one side or other have escaped injury in a most surprising matter. This is markedly different from what we had been led to expect from some of the experiments which had been made upon dead bodies. At any rate, it is found in practice that the tracks of uncomplicated wounds through soft parts caused by Mauser bullets are of very small calibre, and in some cases pick out certain individual structures in a remarkable manner, and also that the apertures of entry and exit are not only both of them extremely small, but when the wound traverses soft parts alone, often differ but little in appearance. In the production of these neat and clean wounds much seems to depend upon the angle at which the bullet impinges upon the surface, and upon the bullet not meeting with anything by which its shape is altered. If the bullet be at all deformed by having struck some hard substance before entering the body, the character of the wound is materially modified, while if, after entering the body, its shape be altered by impinging on a bone, the exit wound will become the large and lacerated wound described by the experimenters and by the older authors. Mr. Makins[1] says that when the bullet impinges on the surface at approximately a right angle, the aperture of entry is circular and clean punched out, about a third of an inch in diameter, and the margin is slightly depressed and contused, while the exit is usually more slit-like, and the surrounding contusion less marked, the size in uncomplicated cases not exceeding that of the aperture of entry, and there being rarely any appearance of starring. When the bullet impinges obliquely the aperture of entry is oval, one side of the entrance-hole sloping down in a shelving manner towards the track, and when such openings are supported by a firm floor, as by the chest walls or other bony substance, they are often of a considerable size. When, however, the shape of the bullet has become altered by striking some hard substance, and when the casing has got torn back so as to form a fringe, or has split so as to form a cutting edge, the apertures of entry and exit lose all special characteristics, becoming large and irregular, and the whole track of the wound becoming modified in a corresponding degree. It is evidently to a large extent this deformation of the bullet, and the accompanying carrying forward of broken fragments of bone, which cause wounds connected with injury of bone to assume such a very different character from the simple flesh wounds spoken of above. But a not inconsiderable number of cases seem to have occurred in which bones as well as soft parts have been simply traversed by the bullets without much corresponding laceration. Much depends upon the velocity of the bullet, and upon the direction in which it impinges on the obstructing bone, matters so purely accidental, that it is no longer possible to lay down general rules, such as are to be found in the older text-book in regard to the characteristics of bullet wounds.

[1] Brit. Med. Jour., Feb. 10.

